# The Accuracy of Tidal Volume Measured With a Smart Shirt During Tasks of Daily Living in Healthy Subjects: Cross-sectional Study

**DOI:** 10.2196/30916

**Published:** 2021-10-18

**Authors:** Denise Mannée, Frans de Jongh, Hanneke van Helvoort

**Affiliations:** 1 Department of Pulmonary Disease Radboud University Medical Centre Nijmegen Netherlands; 2 Department of Engineering Fluid Dynamics University of Twente Enschede Netherlands

**Keywords:** telemonitoring, Hexoskin smart shirt, smart textiles, textile sensors, accuracy, healthy subjects, calibration, tidal volume

## Abstract

**Background:**

The Hexoskin is a smart shirt that can take continuous and objective measurements and could be part of a potential telemonitoring system.

**Objective:**

The aim of this study was to determine the accuracy of the calibrated Hexoskin in measuring tidal volumes (TVs) in comparison to spirometry during various tasks.

**Methods:**

In a cross-sectional study, the TV of 15 healthy subjects was measured while performing seven tasks using spirometry and the Hexoskin. These tasks were performed during two sessions; between sessions, all equipment was removed. A one-time spirometer-based calibration per task was determined in session 1 and applied to the corresponding task in both sessions. Bland-Altman analysis was used to determine the agreement between TV that was measured with the Hexoskin and that measured with spirometry. A priori, we determined that the bias had to be less than ±5%, with limits of agreement (LOA) of less than ±15%. Lung volumes were measured and had to have LOA of less than ±0.150 L.

**Results:**

In the first session, all tasks had a median bias within the criteria (±0.6%). In the second session, biases were ±8.9%; only two tasks met the criteria. In both sessions, LOA were within the criteria in six out of seven tasks (±14.7%). LOA of lung volumes were greater than 0.150 L.

**Conclusions:**

The Hexoskin was able to correctly measure TV in healthy subjects during various tasks. However, after reapplication of the equipment, calibration factors were not able to be reused to obtain results within the determined boundaries.

**Trial Registration:**

Netherlands Trial Register NL6934; https://www.trialregister.nl/trial/6934

## Introduction

Various pulmonary diseases are characterized by exacerbations, which are defined as acute and subacute increases in respiratory symptoms beyond normal variations. These exacerbations may warrant change in regular therapy, and sometimes hospitalization is necessary. Many patients have difficulties perceiving acute exacerbation of chronic obstructive pulmonary disease (AE-COPD) symptoms in a timely manner and, therefore, are not likely to seek consultation for additional therapy [1]. To decrease the unwanted effects of unreported AE-COPD, insight should be gained in the early changes in respiratory parameters and symptoms in the prodrome phase of AE-COPD. Telemonitoring parameters related to breathing provide these insights and could lead to a reduction in exacerbations and hospitalizations [2-4]. Current telemonitoring methods have the following disadvantages: (1) subjective evaluation of symptoms could increase awareness, causing overresponsiveness [5]; (2) measurement of forced expiratory volume in the first second of expiration (FEV_1_) alone does not fully capture the symptoms and the patient’s perception of their health status [6]; and (3) lung function parameters are measured at rest and, therefore, poorly represent the daily problems with physical activities [7]. A potential method without these disadvantages, where measurements can be taken continuously and objectively, is the use of a Hexoskin smart shirt (Carré Technologies). The shirt can be worn by a patient and connected to a “smart device.” The Hexoskin contains respiratory inductance plethysmography (RIP), which consists of a thoracic and abdominal belt. In addition to the RIP sensors, the Hexoskin also contains three electrocardiogram sensors and an acceleration sensor. Various lung parameters can be derived from the signals, such as minute ventilation, respiratory rate, tidal volume (TV), and inspiratory and expiratory times.

The validity of the lung parameters measured by the Hexoskin has been studied by multiple researchers in the last several years. In these studies, it has been shown that the uncalibrated signals obtained with the Hexoskin follow the clinical standard to a good extent [8-10]. There are no studies showing the accuracy of the Hexoskin in measuring spirometer-calibrated TV; however, other studies with another smart shirt, LifeShirt, using RIP technology were found. The no-longer available LifeShirt is a “snuggly” fit garment containing two RIP belts, electrocardiogram sensors, an accelerometer, and a pulse oximeter [11]. Clarenbach et al [11] found no significant bias, and limits of agreement (LOA) were within 8% for TV with qualitative diagnostics calibration. Brüllmann et al [12] used task-specific qualitative diagnostics calibration in lying, standing, and sitting. The bias was zero in all activities, and LOA ranged from 7% to 14%. Hollier et al [13] found a bias of 5 mL (1%) and LOA of 20% in healthy controls. They used fixed-volume calibration while the subject was in the sitting position. The calibration parameters for calculating real volumes were strongly dependent on the position of the belts and on the posture and activity of the subject [12,14,15].

Ideally, the Hexoskin should be able to measure TV accurately during various tasks of daily living, and a patient should be able to remove and reapply the Hexoskin without repeating the calibration procedure. The main focus of our study is, therefore, to determine the accuracy of the calibrated Hexoskin to measure TV in comparison to a spirometer in healthy subjects, before burdening patients with advanced lung disease. As a secondary goal, we investigated whether a one-time spirometry-based calibration could be reapplied after removal and reapplication of the Hexoskin. Moreover, we investigated the possibility of using the Hexoskin to measure functional lung volumes, such as slow vital capacity (SVC), forced vital capacity (FVC), and FEV_1_. If the accuracy for functional lung volumes is good, this would make it possible to combine all measurements during home monitoring into one device. Lastly, we questioned the comfort of our subjects while wearing the Hexoskin.

## Methods

### Subjects

The Hexoskin study was performed between October 2018 and February 2019, as part of a multicenter, cross-sectional study. The study was registered with the Netherlands Trial Register (NL6934) and was approved by the Twente Medical Ethics Committee. A total of 15 subjects without known pulmonary or cardiac disease and with normal pulmonary function (ie, FEV_1_>80% of predicted), between the ages of 18 and 80 years, were recruited at the University of Twente, Enschede, the Netherlands. Subjects were excluded when they were (1) not able to perform the tests, (2) not able to fit into one of the available Hexoskin sizes, (3) had a pacemaker or implantable cardioverter defibrillator, or (4) were unable to read and understand the Dutch language. All subjects gave written informed consent prior to participation.

### Study Design

#### Overview

**Figure 1:**
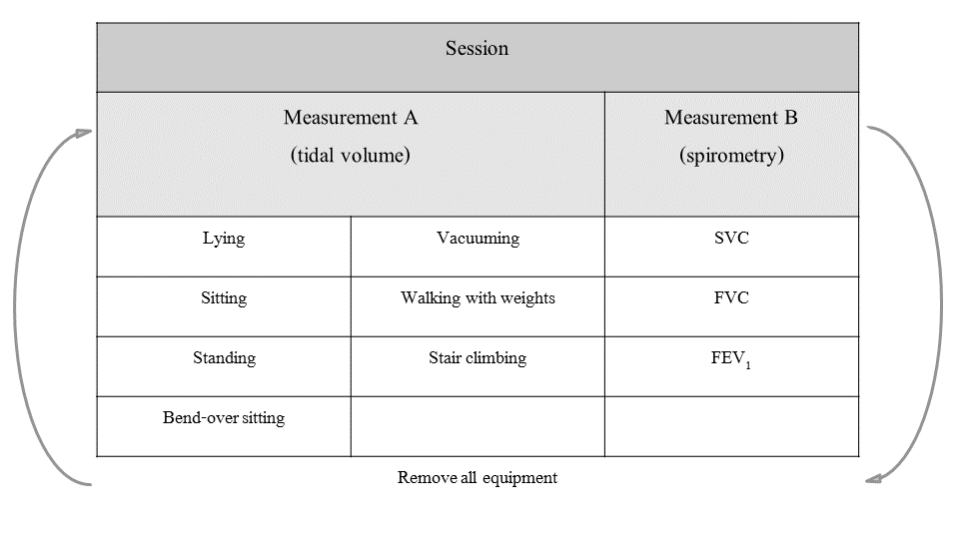
Overview of session and measurements. After a session, all equipment was removed and reapplied. FEV_1_: forced expiratory volume in the first second of expiration; FVC: forced vital capacity; SVC: slow vital capacity.

The circumferences of the thorax and abdomen of a subject, measured according to Carré Technologies’ instructions, were used to select the most proper-sized Hexoskin. The RIP sensors in the Hexoskin measure fluctuations in the cross-sectional area of the chest wall based on changes in inductance, reflecting ventilation of the person wearing the Hexoskin. All subjects wore the Hexoskin for a minimum of 5 minutes before starting with the measurement to get used to the Hexoskin. After this period, they performed two sessions of measurements; see Figure 1 for an overview. Each session contained two measurements: measurement A and measurement B. At the end of session 1, all equipment was removed, including the Hexoskin. Within an hour, all equipment was reattached, and all measurements were repeated in session 2. Afterwards, subjects answered two questions describing their experience with the measurements.

#### Measurement A

During measurement A, seven tasks of daily living were executed. The tasks constituted of 5 minutes each of lying, sitting, standing, bend-over sitting, vacuum cleaning, walking with weights (5 kg), and stair climbing (15 steps up and down). The latter four tasks were based on tasks that cause patients with pulmonary disease to complain of dyspnea in their daily lives [16]. The activities were performed in a pace determined by the subjects. During measurement A, respiration was recorded with the Hexoskin and a calibrated flow sensor from the Oxycon Mobile (Vyaire Medical).

Raw Hexoskin data were obtained from the online database of Carré Technologies. The data were processed with an algorithm made by the author in MATLAB (version 2018a; MathWorks). The data were filtered with a Savitzky-Golay filter to decrease high-frequency noise and movement artefacts. Thoracic and abdominal signals were summed to obtain one signal. Cross-correlation was used to correct for any time delay between the Hexoskin and the spirometer. All data were split into seven pieces, representing the seven tasks. The algorithm determined all end-expiratory and end-inspiratory lung volumes, which were used to calculate TV. TV measured with the Hexoskin (TV_HX_) was calibrated based on TV measured with the spirometer (TV_SPIRO_). All TV values in a task were used to determine calibration factors *a* and *b* with least squares linear regression (see equation 1). This resulted in 15×7 sets of calibration factors.




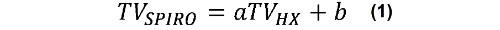




After calibration, an averaging window of 5 TVs was applied to all data to perform further analysis. The seven calibration functions from session 1 were applied to the seven tasks of session 2 to determine accuracy after removal of the Hexoskin.

A Bland-Altman analysis was used to determine the agreement between TV_HX_ and TV_SPIRO_. The difference (diffTV) between the methods was represented as a percentage of TV_SPIRO_:




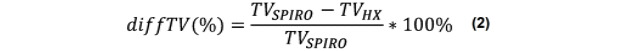




For each task and for each individual, a bias and LOA were derived, and the mean or median bias and LOA for all subjects was determined per task. The definition of good agreement between the methods was established a priori. The median or mean bias had to be between –5% and 5%, and the LOA had to be between –15% and 15%. These criteria were established by clinicians and their opinions on accuracy in monitoring of TVs, based on their experience with cardiopulmonary exercise testing in the hospital.

#### Measurement B

During measurement B, subjects performed spirometry maneuvers according to the European Respiratory Society and American Thoracic Society guidelines [17] for lung function measurements. Lung function was synchronously recorded with the Hexoskin and a pneumotachograph, the Vyntus SPIRO (Vyaire Medical). Lung parameters measured were SVC, FVC, and FEV_1_.

The raw data from measurement B were processed in the same manner as those from measurement A, as seen in the Measurement A section. The calibration function of the “sitting” task in measurement A was used to calibrate the Hexoskin signal to obtain SVC, FVC, and FEV_1_.

To calculate the bias and LOA per session, the SVC, FVC, and FEV_1_ for all subjects were used in Bland-Altman analysis. The bias should be close to zero, and LOA should not exceed ±0.150 L. These criteria were based on the European Respiratory Society and American Thoracic Society statements on lung function measurement [17]. The coefficient of variation was calculated per subject for SVC and FVC by dividing the SD by the mean.

#### Questions on Experience

After the measurements were conducted, we asked all subjects to answer two questions on their experience with the smart shirt. Responses to both questions were answered using a scale from 1 to 10. The first question was “What did you think of the fit of the shirt?”; a response of 1 represented “very loose,” 5 represented “perfect fit,” and 10 represented “very tight.” The second question was “How comfortable were you whilst wearing the shirt?”; a response of 1 represented “not comfortable,” 5 represented “slightly comfortable,” and 10 represented “very comfortable.”

### Statistical Analysis

Baseline characteristics are described by mean (SD) or median (IQR), depending on normality. Normality was assessed by a Shapiro-Wilk test.

## Results

### Overview

A total of 15 healthy subjects were included in this study; no subjects were excluded. Baseline characteristics are presented in Table 1.

**Table 1:** Baseline characteristics of the participants.

Characteristic		Value (N=15)
Age (years), median (IQR)		28 (25-51)
**Sex, n (%)**		
	Male	8 (53)
	Female	7 (47)
Height (cm), median (IQR)		180 (173-188)
Weight (kg), median (IQR)		70 (65-83)
Circumference of thorax (cm), median (IQR)		87 (79-92)
Circumference of abdomen (cm), median (IQR)		85 (76-89)
**Hexoskin size, n (%)**		
	Extra small	3 (20)
	Small	2 (13)
	Medium	5 (33)
	Large	4 (27)
	Extra large	1 (7)

### Measurement A

**Figure 2:**
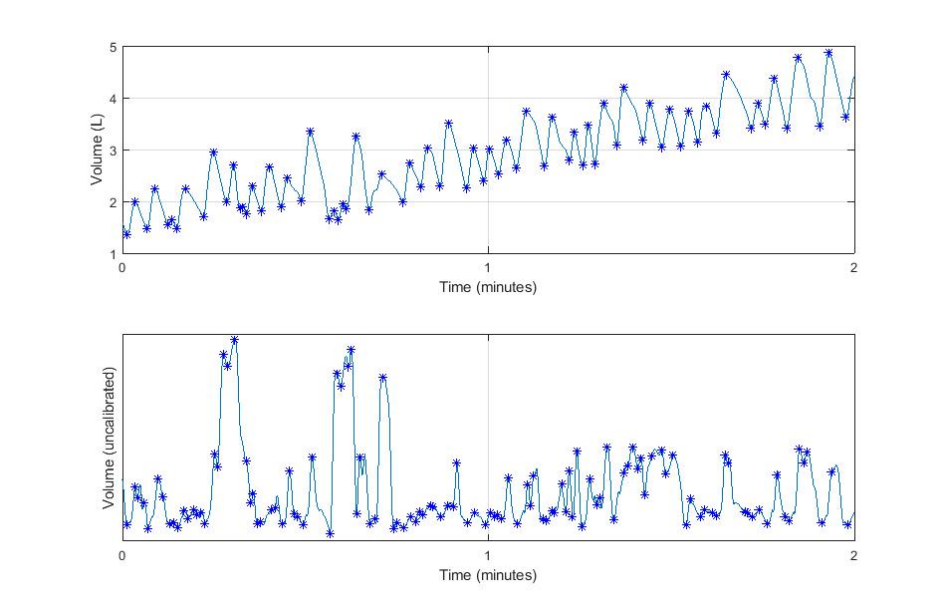
Example of data excluded from further analysis because of extensive artefacts. In the upper plot, 60 seconds of the spirometry data are displayed. In the lower plot, the corresponding Hexoskin data are displayed. The blue stars represent the end-expiratory and end-inspiratory levels used for tidal volume calculation. As can be seen from both panels, in more than 50% of the measurements, it is impossible to find corresponding peaks between the spirometer and the Hexoskin.

In both sessions, out of 15 participants, individuals had to be excluded from further analysis due to artefacts in Hexoskin data regarding the following tasks: bend-over sitting (n=1, 7%), vacuuming (n=3, 20%), walking with weights (n=3, 20%), and stair climbing (n=4, 27%). Data from vacuuming and walking with weights tasks had to be removed from measurements for both subjects 1 and 2. Moreover, subject 2 data also showed large artefacts in the stair climbing task. In total, 50% of the removed data were explained by the removal of data from measurements for two individuals. Regarding the Hexoskin data, TV could not be distinguished from the artefacts and, therefore, we were unable to match TV between the two methods. If more than 50% of the data within a task were unmatchable, they were removed from further analysis. Figure 2 shows an example of the excluded data.

TV was normally distributed. We listed the tasks with the lowest and highest mean TV. In session 1, the mean TV_SPIRO_ ranged from 0.67 (SD 0.14) L for bend-over sitting to 1.57 (SD 0.48) L for stair climbing. The mean TV_HX_ ranged from 0.67 (SD 0.13) L for bend-over sitting to 1.57 (SD 0.46) L for stair climbing. In session 2, the mean TV_SPIRO_ ranged from 0.61 (SD 0.12) L for bend-over sitting to 1.63 (SD 0.49) L for stair climbing. The mean TV_HX_ ranged from 0.61 (SD 0.21) L for lying to 1.69 (SD 0.44) L for stair climbing.

**Figure 3:**
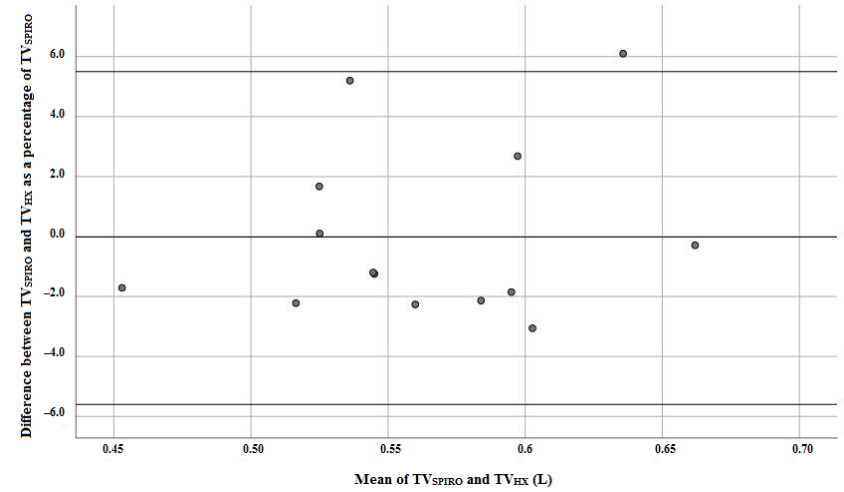
Example of a Bland-Altman plot of tidal volume (TV) of the task "lying" for subject 6. TV_HX_: tidal volume measured with the Hexoskin; TV_SPIRO_: tidal volume measured by spirometry.

With Bland-Altman analysis, the bias and LOA were calculated comparing TV_SPIRO_ and TV_HX_ in each session; see Figure 3 for an example. The biases found in subjects were nonnormally distributed. The median bias (median LOA) for all subjects per task are given in Table 2. In session 1, all tasks, except for vacuuming, met the criteria for the bias and LOA. In session 2, all tasks, except for vacuuming, met the criteria for LOA. Two tasks met the criteria for the bias.

**Table 2:** Bland-Altman analysis was used to calculate the percentage bias (limits of agreement [LOA]) between tidal volume measured with spirometry and tidal volume measured with the Hexoskin.

Session	Task, median % bias (median LOA)						
	Lying (N=15)	Sitting (n=14)	Standing (N=15)	Bend-over sitting (N=15)	Vacuuming (n=12)	Walking with weights (n=12)	Stair walking (n=13)
Session 1	0.0 (5.6)	–0.2 (12.7)	–0.2 (12.0)	–0.4 (10.8)	–0.6 (16.7)	–0.3 (11.1)	–0.5 (12.6)
Session 2	7.5 (6.7)	–6.4 (14.7)	–8.5 (13.0)	3.3 (10.6)	–8.9 (16.4)	–7.3 (12.0)	–4.5 (15.0)

### Measurement B

**Figure 4:**
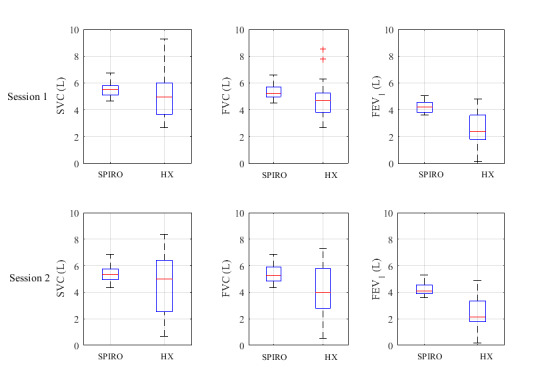
Box plots of all lung function parameters (n=30). FEV_1_: forced expiratory volume in the first second of expiration; FVC: forced vital capacity; HX: Hexoskin; SPIRO: spirometer; SVC: slow vital capacity.

Due to excessive movement artefacts, as a result of breathing maneuvers, data from 5 subjects in the Hexoskin group had to be excluded from further analysis in both sessions. All lung parameters were normally distributed. A box plot of all functional lung parameters can be found in Figure 4.

**Figure 5:**
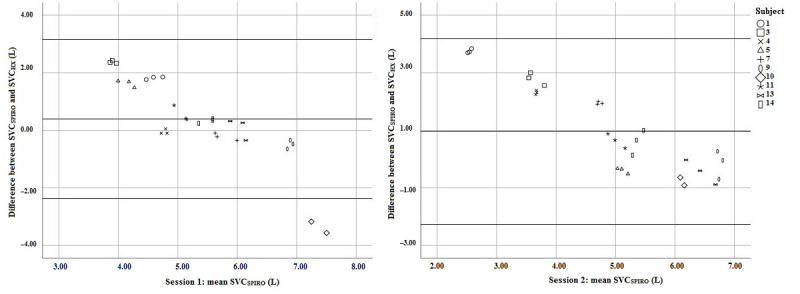
Bland-Altman plots of slow vital capacity (SVC) (n=30). HX: Hexoskin; SPIRO: spirometer.

With Bland-Altman analysis, the bias and LOA were calculated by comparing the various lung function parameters measured with the spirometer and the Hexoskin. The bias (LOA) of the SVC, FVC, and FEV_1_ for each session can be found in Table 3. All Bland-Altman plots have similar features; see Figure 5 for Bland-Altman plots of SVC. Trends were found in all Bland-Altman plots. None of the parameters met the criteria for accuracy, and Hexoskin data showed large deviations from spirometry data. The coefficient of variation was smaller than 10% for SVC and FVC measured with both methods.

**Table 3:** Bland-Altman analysis was used to calculate the bias (limits of agreement [LOA]) between various lung parameters (volume_spirometer_–volume_Hexoskin_) in measurement B. Bias and LOA were based on 30 measurements (measurement of lung parameters in triplicate in 10 subjects).

Session	Lung parameter, bias (LOA)		
	Slow vital capacity, L	Forced vital capacity, L	Forced expiratory volume in the first second of expiration, L
Session 1	0.39 (2.76)	0.68 (2.23)	1.73 (2.20)
Session 2	0.96 (3.23)	1.26 (2.72)	1.85 (1.82)

### Questions on Experience

All subjects answered both questions. The mean rating for the fit of the shirt was 6 (SD 1). Comfort had a mean score of 8 (SD 2).

## Discussion

### Principal Findings

In this study, we determined the accuracy of the calibrated Hexoskin to measure TV in comparison to the spirometer in various tasks of daily living. In all tasks of session 1, accuracy of the Hexoskin was good, with biases within the criteria and acceptable LOA. Moreover, reapplicability of the calibration functions was tested in a repeated session. The high median bias in the second session showed that a recalibration was necessary after removal of the Hexoskin. However, LOA were comparable with the first session, which indicates that there was only an accuracy issue and not a precision problem. The Hexoskin is not yet a valid measurement tool to measure SVC, FVC, and FEV_1_, based on bias and LOA. Lastly, the comfort level of subjects was high while wearing the Hexoskin.

### Accuracy of TV Measurement During Activities of Daily Living

A priori, we determined that for good accuracy, the bias had to be between –5% and 5%, and the LOA had to be between –15% and 15%. Good accuracy of the Hexoskin in measuring TV was found in the first session. The median bias was found to be within ±0.6%, comparable to a bias of 3 mL on a TV of 0.500 L. Moreover, all LOA were within the ±15% that we determined a priori, with one exception for vacuuming (15.6%). The median biases were all negative, indicating that the Hexoskin overestimated TV slightly, on average.

The LOA found in the various tasks were slightly lower than the LOA in the previously mentioned studies [12,13], with the exception of the study by Clarenbach et al [11]. We hypothesize that the slight variations in outcome were the result of the performed analysis, in following three ways:

1. Calibration time. We used a calibration time of 5 minutes and Clarenbach et al [11] used 1 minute. It is hypothesized that the calibration is more accurate with longer calibration time and LOA are lower.
2. Bland-Altman analysis. We calculated a median bias over all subjects, while in all other studies the bias was calculated over all subjects. If the bias slightly varies among subjects, LOA will increase when one Bland-Altman analysis is performed.
3. Averaging window. We used an averaging window of 5 breaths; Clarenbach, for example, used 20 breaths. This could have increased the agreement, as a larger averaging window tends to decrease variations in RIP.

More demanding tasks (ie, bend-over sitting, vacuuming, walking with weights, and stair climbing) showed a slightly decreased agreement relative to the tasks at rest. The decrease in agreement was also reflected in the data we had to remove due to excessive artefacts; they were all part of a demanding task. Similarly, in the study by Brüllmann et al [12], agreement was decreased more highly in demanding tasks compared to agreement in a resting position. The increase in LOA from inactivity to more demanding tasks can be explained by the increase (1) in variation in breathing patterns [18,19] and (2) of movement artefacts.

### Reapplicability of Calibration Factors

In session 2, we investigated the possibility of reusing calibration factors. Although the median biases were relatively small, in five out of seven tasks they did not meet the criteria. The LOA in the second session were comparable to the LOA found in session 1 in all tasks, with vacuuming as the exception, within 15%. This is in contrast with earlier studies. Heyde et al [20] found that the bias between RIP and the spirometer was similar in repeated measurements. Moreover, Brüllmann et al [12] compared calibration parameters between two measurements and found that the mean difference was zero.

Various reasons for poor accuracy in session 2 can be distinguished, based on poor calibration. The position and fit of the RIP belts on the subjects affect the calibration needed to get accurate results [12,14,15]. Various subjects were in between Hexoskin sizes; because of this, during measurements with these subjects, the Hexoskin tended to crawl up. Therefore, the chance is high that the Hexoskin and, thus, the thoracic and abdominal bands slightly changed positions (1) during measurements and (2) in between measurements. A possible solution to this problem is presented in the study by Brüllmann et al [12]. The LifeShirt had a snug fit but was also affixed to the trousers of the subject with pins to prevent the upward movement of the shirt. Another explanation for not being able to reapply the calibration factors is magnetic coupling of the two belts in the Hexoskin. This appears to occur due to the mutual inductance between chest and abdominal bands modulating the output of the shirt. This is a known problem of RIP sensors [21]. The chance of poor reproducibility is high, as the influence of the bands on each other is not predictable when reapplying the Hexoskin.

### Measurement of Functional Lung Parameters

A priori, we determined that all lung volumes should be measured with a bias close to zero and LOA equal to or smaller than 0.150 L. None of the lung volumes were measured within these criteria. To the knowledge of the authors, there have been no studies published investigating the feasibility of RIP technology to measure functional spirometry. Poor accuracy in this study can be explained by (1) movement of the shirt, (2) assumption of a linear relationship between circumference and lung volumes, and (3) the use of calibration factors determined from the “sitting” task. The first explanation has already been addressed in the previous section. The second and third argument are related. Based on earlier studies [22], the assumption was made that there is a linear relationship between circumference and lung volumes. However, this assumption is probably true for normal or resting breathing and not for forced maneuvers. We used the calibration of the “sitting” task. In this task, the volumes exhaled were, on average, 0.7 L, while in SVC and FVC maneuvers, volumes above 5 L, on average, were exhaled. This could explain the large deviation between volumes measured by RIP and the spirometer. The linear relationship between the mean and the difference in the Bland-Altman plots (Figure 5) can be explained by the difference in distribution of volumes (Figure 4) measured with both the Hexoskin and the spirometer.

Although we cannot determine the bias and LOA per subject, this would be interesting. Per subject, all SVC and FVC values measured with the Hexoskin seemed to be in very close range to each other (Figure 5). The mean coefficient of variation was comparable in both techniques. For SVC and FVC, the coefficient of variation was <10% for both techniques. This suggests that it is possible to measure functional lung volumes accurately; however, a proper calibration procedure should be defined containing a set with variations in lung volumes (eg, 200 mL to 2000 mL).

### Limitations

In this study, we identified some limitations. In session 1, the same data were used for determination and validation of the calibration factors. We used this construction to minimize the measurement time for the subjects, and to make the calibration procedure realistic and applicable in a clinical setting for patients with pulmonary diseases. However, if the calibration varies over time within a task without removal of the equipment, we should have found larger LOA. Therefore, it is unlikely that the bias found in this study would change to a significant extent if extra validation data were to be included.

In measurement B, the use of the calibration from the “sitting” task could be the reason for poor accuracy of the measured lung volumes. Accuracy could have been decreased if calibration factors were derived from a sample of breaths larger than 70% of SVC or FVC.

### Clinical Insights

We conclude that the Hexoskin accurately measures TV compared to spirometry values if the calibration procedure is used prior to measurement, as was the case in session 1. Based on our analysis, we conclude that 95% of the measured TV_HX_ values were accurately measured compared to TV_SPIRO_. The other 5% of the measurements will not affect outcomes in home monitoring. During telemonitoring, it is common to measure over hours or days. In such a time span, the outliers will not have a significant impact on the average measurement of TV.

However, the implementation of the shirt as a telemonitoring device is not yet feasible. Currently, based on the results in session 2, calibrations have to be performed prior to each measurement. Due to the long calibration time (approximately 30 minutes), this would not be a practical procedure. Modifications to the shirt to deal with the problems of magnetic coupling will probably solve problems with the nonreproducibility of the calibration. Other solutions are to (1) investigate other calibration procedures that are simpler or shorter in nature or (2) look at uncalibrated data. We suggest, for example, a fixed-volume calibration [15], in which a subject breathes from a known volume for several minutes to obtain the calibration factors. In this case, spirometry measurements become unnecessary.

The shirt can be worn with a high level of comfort during measurements. This is a clinically relevant conclusion, as use of the shirt would not be feasible for patient monitoring if the comfort level was rated lower. Moreover, the manufacturer introduced a zipper into the shirt. This will make use of the shirt even more feasible for patient monitoring, as it will make putting on the shirt easier.

### Conclusions

The spirometer-calibrated Hexoskin was able to accurately measure TV in healthy subjects during various tasks; however, calibration factors could not be used after removal and reapplication of the Hexoskin. The Hexoskin cannot be used to assess calibrated lung volumes reliably. In the near future, we will evaluate other calibration options to improve the reproducibility of the calibration. Moreover, we will determine the accuracy in patients and investigate the usability of the Hexoskin for home monitoring of patients with pulmonary disease.

## Abbreviations

AE-COPD: acute exacerbation of chronic obstructive pulmonary disease
FEV_1_: forced expiratory volume in the first second of expiration
FVC: forced vital capacity
LOA: limits of agreement
RIP: respiratory inductance plethysmography
SVC: slow vital capacity
TV: tidal volume
TV_HX_: tidal volume measured with the Hexoskin
TV_SPIRO_: tidal volume measured with spirometry
